# Management of delivery of a fetus with autosomal recessive polycystic kidney disease: a case report of abdominal dystocia and review of the literature

**DOI:** 10.1186/s13256-019-2293-3

**Published:** 2019-12-12

**Authors:** Sarah Belin, Cristina Delco, Paloma Parvex, Sylviane Hanquinet, Siv Fokstuen, Begoña Martinez de Tejada, Isabelle Eperon

**Affiliations:** 1 Department of Pediatrics, Gynecology and Obstetrics, University Hospitals of Geneva, rue Gabriel-Perret-Gentil 14, 1205 Geneva, Switzerland; 2 Service of Radiology, Department of Diagnosis, University Hospitals of Geneva, rue Gabriel-Perret-Gentil 14, 1205 Geneva, Switzerland; 3 Service of Genetic Medicine, Department of Diagnosis, University Hospitals of Geneva, rue Gabriel-Perret-Gentil 14, 1205 Geneva, Switzerland; 4 Faculty of Medicine, University of Geneva, University Hospitals of Geneva, rue Gabriel-Perret-Gentil 14, 1205 Geneva, Switzerland

**Keywords:** Abdominal dystocia, Autosomal recessive renal polycystic kidney disease, Delivery, Management

## Abstract

**Background:**

Autosomal recessive renal polycystic kidney disease occurs in 1 in 20,000 live births. It is caused by mutations in both alleles of the *PKHD1* gene. Management of delivery in cases of suspected autosomal recessive renal polycystic kidney disease is rarely discussed, and literature concerning abdominal dystocia is extremely scarce. We present a case of a patient with autosomal recessive renal polycystic kidney disease whose delivery was complicated by abdominal dystocia, and we discuss the factors that determined the route and timing of delivery.

**Case presentation:**

A 23-year-old Caucasian woman, G2 P0, with a prior unremarkable pregnancy was referred to our tertiary center at 31 weeks of gestation because of severe oligoamnios (amniotic fluid index = 2) and hyperechogenic, dedifferentiated, and enlarged fetal kidneys. She had no other genitourinary anomaly. Fetal magnetic resonance imaging showed enlarged, hypersignal kidneys and severe pulmonary hypoplasia. We had a high suspicion of autosomal recessive renal polycystic kidney disease, and after discussion with our multidisciplinary team, the parents opted for conservative care. Ultrasound performed at 35 weeks of gestation showed a fetal estimated weight of 3550 g and an abdominal circumference of 377 mm, both above the 90th percentile. Because of the very rapid kidney growth and suspected risk of abdominal dystocia, we proposed induction of labor at 36 weeks of gestation after corticosteroid administration for fetal lung maturation. Vaginal delivery was complicated by abdominal dystocia, which resolved by continuing expulsive efforts and gentle fetal traction. A 3300-g (P50–90) male infant was born with Apgar scores of 1-7-7 at 1, 5, and 10 minutes, respectively, and arterial and venous umbilical cord pH values of 7.23–7.33. Continuous peritoneal dialysis was started at day 2 of life because of anuria. Currently, the infant is 1 year old and is waiting for kidney transplant that should be performed once he reaches 10 kg. Molecular analysis of *PKHD1* performed on deoxyribonucleic acid (DNA) from the umbilical cord confirmed autosomal recessive renal polycystic kidney disease.

**Conclusions:**

Management of delivery in cases of suspected autosomal recessive renal polycystic kidney disease needs to be discussed because of the risk of abdominal dystocia. The route and timing of delivery depend on the size of the fetal abdominal circumference and the gestational age. The rate of kidney growth must also be taken into account.

## Background

Autosomal recessive renal polycystic kidney disease (ARPKD) is a rare form of cystic kidney disease, occurring in approximately 1 in 20,000 live births [1]. It is caused by mutations in the *PKHD1* (polycystic kidney and hepatic disease 1) gene, situated on chromosome 6p12, which encodes for the protein fibrocystin [2]. It has an autosomal recessive mode of inheritance, and most affected children are compound heterozygotes, inheriting a different ARPKD mutation from each parent [3]. Phenotype is variable, with up to 30% of cases dying in the neonatal period or within the first year of life, presenting *in utero* with severe oligohydramnios leading to Potter’s sequence with pulmonary hypoplasia, characteristic facies, and limb deformations [4, 5]. In less severe presentations, prognosis is difficult to establish prenatally and has been shown to correlate with the amount of amniotic fluid and lung volume calculated on the basis of fetal magnetic resonance imaging (MRI) [6, 7]. Prenatal counseling and clinical management are best conducted by multidisciplinary care teams that include obstetricians, neonatologists, pediatricians specialized in nephrology, radiologists, and geneticists [3]. We present a case of a pregnant woman with suspected fetal ARPKD diagnosed at 31 weeks of gestation and discuss the elements orienting pregnancy and delivery management.

## Case presentation

**Fig. 1:**
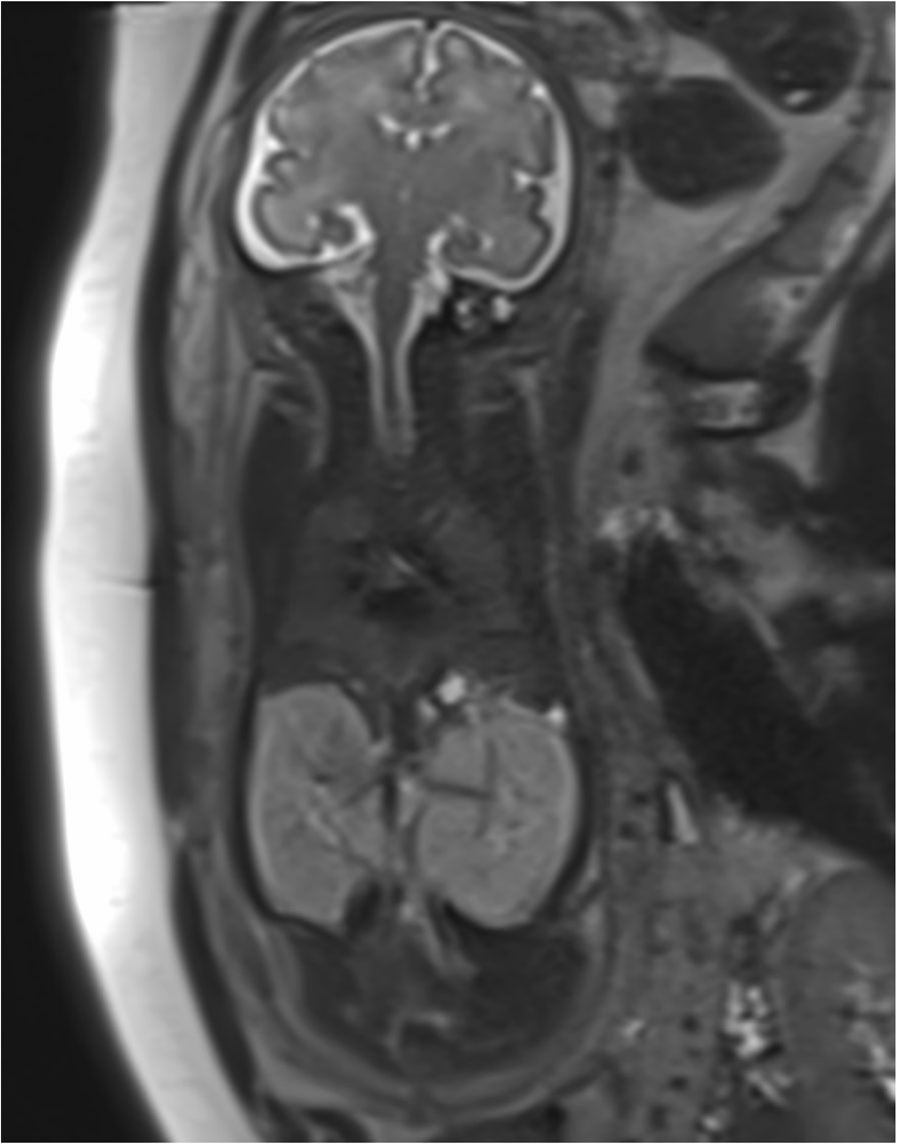
Fetal magnetic resonance imaging at 31 weeks of gestation. Coronal T2 image shows abdominal cavity occupied by two large hyperechogenic kidneys (5.5 cm) and decreased thorax size

We present a case of a spontaneous pregnancy in a healthy nonconsanguineous couple with an unremarkable family history. The mother was 23-year-old and had a history of a voluntary pregnancy termination and active tobacco use (five to eight cigarettes per day). She underwent regular pregnancy follow-up. First-trimester screening included an ultrasound (US) scan with a thin nuchal translucency (1.4 mm) and a very low risk for trisomy 21 (1 in 19,000). Blood tests showed immunity against cytomegalovirus (immunoglobulin G [IgG]-positive, IgM-negative), and a US scan at 20 weeks of gestation showed no signs of fetal anomalies and a normal amount of amniotic fluid. A US scan at 28 weeks of gestation showed a slight reduction with an amniotic fluid index (AFI) of 7 and a deepest pocket of 2.3 cm. At 31 weeks of gestation, the patient was referred to our tertiary fetal medicine unit because of severe oligoamnios and enlarged kidneys. We confirmed the presence of severe oligoamnios (AFI = 2) as well as hyperechogenic, undifferentiated, and enlarged bilateral fetal kidneys (right side, 39 × 64 × 25 mm; left side, 33 × 63 × 39 mm; >97th percentile [8]). There was no ectasia of the urinary tract. The bladder was visualized but only slightly filled, and no spontaneous micturition was seen. The male genital organs of the fetus were without anomalies. Estimated fetal weight was above the 90th percentile at 2240 g. Head circumference was 296.5 mm (60th percentile), and abdominal circumference was 300.6 mm (>95th percentile). Maternal kidneys were normal. At 31 weeks of gestation, a fetal MRI was performed and showed two enlarged kidneys (length, 6 cm; normal mean, 3 cm at this gestational age) with high signal intensity on T2-weighted images and loss of corticomedullary differentiation, a microbladder, severe oligoamnios, and a small chest with lung volumes of 15 and 17 cm^3^, respectively (normal mean total lung volume [TLV] at 31 weeks, 65 cm^3^ [9]) (Fig. 1). We suspected ARPKD, with poor prognosis due to the severe oligoamnios and small pulmonary volumes. After discussion in a multidisciplinary team with the neonatologists and pediatric nephrologists, the couple opted for conservative care.

**Fig. 2:**
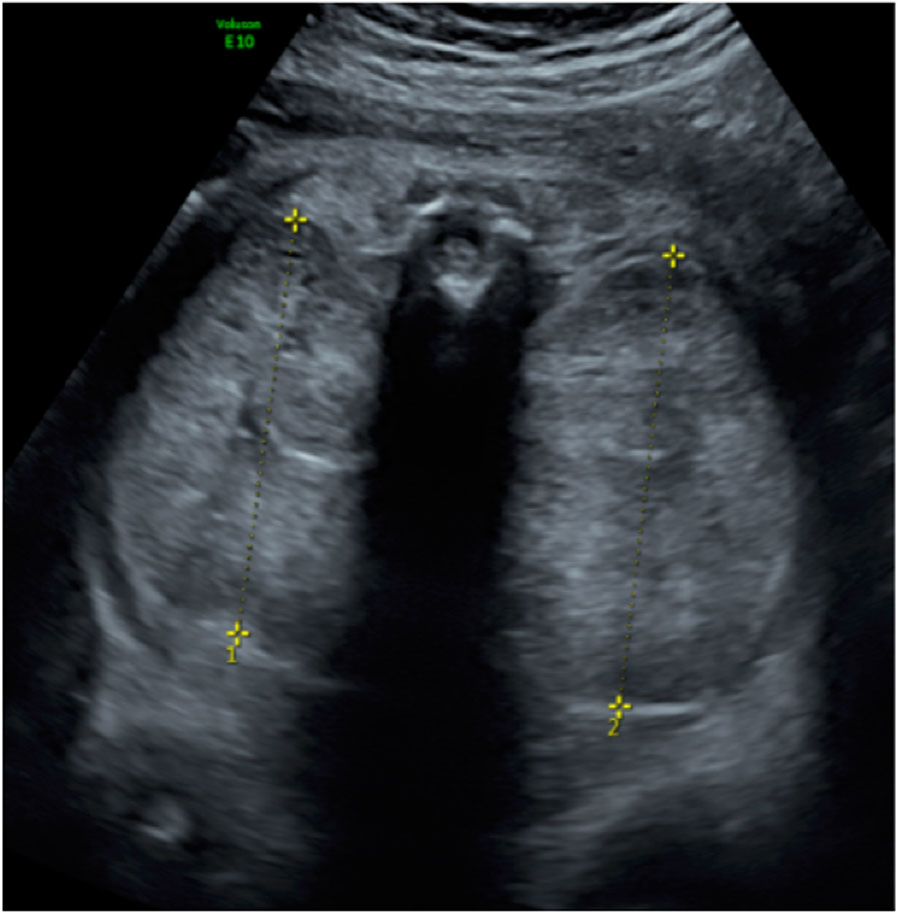
Ultrasound at 35 weeks of gestation. Right kidney measures 65 × 90 × 63 mm; left kidney measures 70 × 78 × 75 mm

**Fig. 3:**
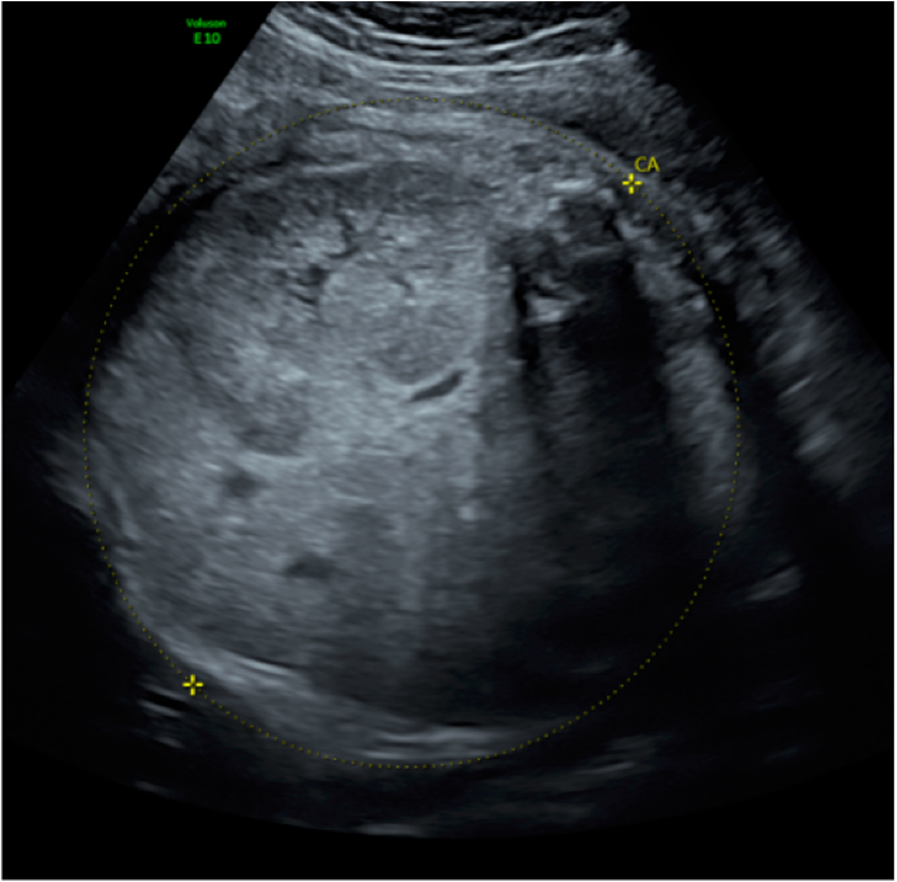
Ultrasound at 35 weeks of gestation with abdominal circumference measurement

US follow-up at 35 weeks of gestation showed a fetal estimated weight of 3550 g and an abdominal circumference of 377 mm, both >97th percentile (Figs. 2 and 3). The head circumference was 318 mm (35th percentile). There was no amniotic fluid. Because of rapid kidney growth and risk of abdominal dystocia, we proposed induction of labor at 36 weeks of gestation after corticosteroid administration for fetal lung maturation. Vaginal delivery was complicated by abdominal dystocia that was resolved by continuing expulsive efforts and gentle fetal traction. We had decided in advance to perform routine episiotomy, and there were no other perineal tears and no maternal postpartum complications. A male infant was born with Apgar score of 1-7-7 at 1, 5, and 10 minutes, respectively, and arterial and venous umbilical cord pH values of 7.23 and 7.33. The infant’s birth weight was 3300 g (P50–90), height 52 cm (P50–90), and head circumference 32 cm (P10–50). Due to worsening respiratory failure at 2 hours of life, with evidence of pulmonary hypertension as well as lung-restrictive physiology secondary to abdominal distention, the infant was intubated and started on high-frequency oscillation ventilation. He was anuric since birth, and continuous peritoneal dialysis was started on day 2 of life. Extubation was achieved at 1 month of life. The child required surgery, with left nephrectomy performed at 1 month and right nephrectomy 3 weeks later. He was discharged from the hospital at 6 months of life. At that time, he showed a slight delay in acquisitions for his age but with adequate interaction with people and the environment. Concerning his motor skills in particular, he was able to maintain his head in axis when lying on his back, to sustain a sitting position helped by lumbar support (but with some discomfort due to his distended abdomen) and to hold objects for a short amount of time when they were placed in his hand. Currently, he is 12 months old and requires peritoneal dialysis while waiting for a kidney transplant that should be performed at the age of 2–3 years.

Molecular analysis performed after birth on deoxyribonucleic acid (DNA) extracted from the umbilical cord confirmed ARPKD, with two heterozygote deletions causing a shift in the reading frame of the *PKHD1* gene. The mother carries c.10035_10044del, p. (Cys3346Glnfs*51), and the father carries c.4485del, p.(Ser1496Valfs*4).

## Discussion

We present a case of a pregnant woman with suspected fetal ARPKD diagnosed at 31 weeks of gestation in which the parents opted for conservative care. Vaginal delivery was elected; induction of labor was proposed at 36 weeks of gestation because of rapid kidney growth; and delivery was complicated by abdominal dystocia. This case is particularly interesting because there is little available literature regarding abdominal dystocia, and both the route and timing of delivery are rarely discussed in existing articles concerning cases of ARPKD.

ARPKD presents prenatally with visualization of bilateral hyperechogenic enlarged kidneys with poor corticomedullary differentiation on second- or third-trimester US scans [1, 3]. There is usually no extrarenal manifestation despite the presence of a Potter’s sequence [1]. In our patient’s case, the clinical presentation was therefore quite typical of ARPKD.

Because there is a large spectrum of phenotypes with varying severity, prognosis is difficult to establish during pregnancy, and prenatal counseling is challenging [10]. Nevertheless, prognosis is usually considered poor, and the rate of pregnancy termination is high.

The infant’s long-term outcome depends mainly on residual renal function and the occurrence of complications due to chronic renal insufficiency and dialysis (until renal transplant). Hypertension can have an important impact and occurs in up to 75% of patients with ARPKD [3, 5, 11]. The long-term morbidity of the disease can also be due to the progressive development of periportal congenital hepatic fibrosis, with associated portal hypertension [12, 13]. However, these elements cannot be determined before birth, and the 5-year survival rate is approximately 90% for neonates who survive the first month of life [11, 14].

Short-term prognosis is determined mainly by respiratory function at birth and the feasibility of neonatal resuscitation [12, 15]. Severe oligohydramnios is considered an indicator of poor prognosis due to the high risk of associated pulmonary hypoplasia [7, 10]. Zaretsky *et al*. suggested the calculation of lung volumes using fetal MRI as an alternative to evaluate the presence of pulmonary hypoplasia and better estimate the prognosis [7]. In their study, the use of a ratio, TLV/gestational age, was comparable to the presence or absence of oligohydramnios as a prognostic tool after 26 weeks of gestation, with a ratio <0.9 indicating a higher risk of nonsurvival. In our patient’s case, severe oligohydramnios was present but was of late onset. Lung volumes on fetal MRI were extremely reduced, prompting a high suspicion of pulmonary hypoplasia. However, using Zaretsky *et al*.’s criteria, the TLV/gestational age ratio was 1.03, thus above the cutoff determining nonsurvival.

Prognosis evaluation should be discussed in a multidisciplinary team considering not only radiological evaluation on MRI but also dynamic US evaluation of kidney growth as well as timing of oligohydramnios onset. Neonatal adaptation to extrauterine life can thus be better anticipated; couples can make an informed decision; and resuscitation and management at birth can be better planned by neonatologists for couples who opt for conservative care, such as in our patient’s case.

Although abdominal dystocia is a major risk influencing obstetric care, management of delivery in cases of suspected ARPKD is rarely discussed. Available literature concerning ARPKD does not focus on obstetric care at delivery, with most series being pediatric retrospective studies not describing the mode of delivery or peripartum obstetric complications. Furthermore, literature concerning abdominal dystocia is extremely scarce, with very few case reports available, most of them very old [16–20]. Among available articles, there are different reported etiologies of abdominal dystocia. Depending on the cause, possible management differs. In cases of meconial peritonitis or ascites, fetal abdominal puncture can be performed to reduce abdominal volume and therefore prevent or resolve abdominal dystocia. This is not possible in cases of ARPKD, because macrocysts are not usually present, and abdominal volume is due to incompressible kidney solid tissue. Therefore, values of abdominal circumference found in the literature that seems to be associated with abdominal dystocia (400 mm of abdominal circumference [21]) have to be interpreted with caution.

In cases of increased abdominal circumference due to suspected ARPKD, mode of delivery needs to be discussed. Abdominal dystocia can also complicate a cesarean delivery. Furthermore, because short-term prognosis is dependent on respiratory function, the benefit–risk equation must take into account the possible adverse effects of induced prematurity versus the possible negative impact of waiting longer, with increasing kidney volumes causing increasing mechanical compression and possibly complicating even more mechanical ventilation at birth.

In our patient’s case, serial US scans repeated at 33 and 35 weeks of gestation showed rapid growth of kidneys with a doubling of kidney size every 2 weeks. At the last US scan at 35 weeks of gestation, we noted an abdominal circumference superior to the 95th percentile of head circumference at 42 weeks of gestation [22]. We opted for induction of labor after fetal lung maturation and to perform routine episiotomy to avoid abdominal trauma. We faced abdominal dystocia that was resolved by continuing gentle traction along the axis during maintained expulsive efforts.

## Conclusion

ARPKD is a severe renal cystic disease that requires multidisciplinary management. Prenatal counseling should be given after discussion between the obstetrical and neonatal teams, pediatric nephrologists, geneticists, and radiologists. An incorporative management is necessary not only to better estimate the short- and long-term prognosis but also to organize maternal and neonatal care, both to maximize efficiency and to prepare the parents for what can be expected. Management of delivery must be discussed because of the risk of abdominal dystocia. Route and timing of delivery depend on the size of the fetal abdominal circumference, the gestational age, and the rate of kidney growth. We recommend performing episiotomy to avoid abdominal trauma.

## Data Availability

All data generated or analyzed during this study are included in this published article.

## Abbreviations

AFI: Amniotic fluid index
ARPKD: Autosomal recessive renal polycystic kidney disease
MRI: Magnetic resonance imaging
*PKHD1*: Polycystic kidney and hepatic disease 1 gene
TLV: Total lung volume
US: Ultrasound
